# The role of ATP bioluminescence in monitoring surface hygiene in hospital settings: a comprehensive review

**DOI:** 10.1186/s13756-026-01698-8

**Published:** 2026-02-21

**Authors:** Ourania S. Kotsiou, Evdoxia Gouta, Nikolaos Natsaridis, Georgios Papageorgiou, Zoe Daniil, Konstantinos I. Gourgoulianis

**Affiliations:** 1 Laboratory of Human Pathophysiology, Department of Nursing, University of Thessaly, 41500 Larissa, Greece; 2 Respiratory Medicine Department, University of Thessaly, 41110 Larissa, Greece; 3 ProGnosis Biotech SA, Farsalon 153, 41335 Larissa, Greece

**Keywords:** ATP bioluminescence, Hospital hygiene, Infection control, Surface monitoring, Environmental cleaning, Healthcare-associated infections (HAIs), Microbial contamination

## Abstract

**Background and objectives:**

Maintaining high standards of environmental hygiene in healthcare settings is critical for preventing healthcare-associated infections (HAIs). Traditional cleanliness assessments, including visual inspections and microbial cultures, often lack sensitivity and immediacy. This review aims to evaluate the utility, advantages, and limitations of adenosine triphosphate (ATP) bioluminescence assays as a rapid, objective method for monitoring hospital surface hygiene.

**Materials and methods:**

A comprehensive review of studies published between 2020 and 2025 was conducted using PubMed and Scopus databases. Studies were included if they evaluated ATP bioluminescence as a primary method for assessing hygiene in clinical environments, medical instruments, or healthcare-related settings.

**Results:**

ATP bioluminescence has demonstrated consistent advantages across diverse healthcare settings. It identifies organic residues undetectable by visual inspection and supports real-time corrective actions, particularly in intensive care units, endoscopy suites, and operating rooms. It has also been effectively used in outbreak investigations, veterinary clinics, and pediatric dental settings. Despite its strengths, ATP assays exhibit variability depending on detector type, surface characteristics, and environmental conditions. Moreover, ATP cannot differentiate microbial from non-microbial sources, necessitating complementary methods for pathogen-specific detection.

**Conclusions:**

ATP bioluminescence is a practical, rapid, and increasingly validated tool for improving surface hygiene monitoring and infection control in healthcare environments. While not a replacement for microbial cultures, it serves as a valuable adjunct to existing assessment methods. Standardized protocols, calibrated thresholds, and integration with other diagnostic tools are essential for maximizing its effectiveness and comparability across institutions.

## Introduction

Maintaining a hygienic hospital environment is critical for patient safety and infection control. Healthcare-associated infections (HAIs) remain a significant challenge worldwide, contributing to increased morbidity, mortality, and healthcare costs. Ensuring cleanliness of frequently touched surfaces, reusable medical instruments, and clinical environments such as intensive care units (ICUs) is an integral part of comprehensive infection prevention strategies [1, 2].

Traditional methods for assessing cleanliness have primarily relied on visual inspections and microbial culture techniques. While these approaches provide valuable data, they are often time-consuming, subjective, and may not capture the full scope of organic contamination present on surfaces. Visual assessments cannot reliably detect microbial or organic residues, and cultures may miss non-culturable yet viable microorganisms, delaying feedback necessary for real-time correction [3, 4].

To address these limitations, ATP bioluminescence assays have been introduced as a rapid, objective method for evaluating surface hygiene. ATP, a molecule present in all living cells, serves as a universal biomarker for organic material. The principle of ATP bioluminescence is based on the enzymatic reaction of luciferase, which emits light proportional to the ATP present on a surface. The resulting relative light units (RLUs) provide an immediate estimate of biological contamination [5, 6].

ATP bioluminescence is increasingly used in various healthcare settings, including hospitals, dental practices, and veterinary clinics. Its application extends from assessing terminal room cleaning in ICUs to validating instrument reprocessing protocols. As a result, infection prevention teams and environmental services departments have integrated ATP monitoring into routine audits and staff training programs to enhance cleaning performance and compliance [7–9].

Despite its advantages, ATP bioluminescence also has limitations. It cannot differentiate between microbial and non-microbial sources of ATP, and surface characteristics or residual disinfectants may affect readings. Moreover, variability among commercial systems and lack of standardized threshold values can challenge interpretation. Therefore, understanding the current evidence on ATP bioluminescence’s effectiveness and its practical implications is essential for informed implementation in healthcare facilities [10–12].

The aim of this review was to critically examine the current literature on the application of ATP bioluminescence in healthcare settings for surface hygiene monitoring. By synthesizing findings from a wide range of studies, this review aims to inform best practices and future research directions for integrating ATP monitoring into hospital hygiene protocols.

## Methods

A systematic review of literature published between 2020 and 2025 was conducted using databases such as PubMed and Scopus. The search strategy included combinations of keywords such as “ATP bioluminescence,” “surface hygiene,” “hospital cleaning,” “infection control,” and “environmental monitoring.” Inclusion criteria were studies that evaluated ATP bioluminescence as a tool for monitoring hygiene on hospital surfaces, medical instruments, or other clinical settings. Excluded were non-peer-reviewed studies, and those not employing ATP assays as a primary assessment method.

## Results

### Application in general hospital surfaces

Mitchell et al. [1] conducted a large-scale observational study across 11 Australian hospitals as part of the REACH trial to evaluate the effectiveness of ATP bioluminescence in monitoring hospital surface hygiene. Over 1200 frequently touched surfaces, such as bed rails, call bells, and over-bed tables, were sampled using ATP assays. The study revealed substantial inter-facility variability, with nearly 40% of surfaces exceeding the recommended ATP thresholds despite having passed visual inspections. These results highlighted the persistence of organic material on high-touch points, indicating potential residual contamination on high-touch surfaces and underscoring the limitations of visual assessment alone [1].

The study further demonstrated that integrating ATP monitoring into routine cleaning protocols enabled real-time detection of hygiene deficiencies. This immediate feedback allowed for targeted training and corrective interventions, leading to measurable improvements in surface cleanliness. The findings provided strong support for the use of ATP bioluminescence as a standardized, objective tool to enhance environmental hygiene and infection prevention practices in general hospital settings [1].

Kajigaya et al. [13] offered additional evidence by investigating ATP contamination levels on doorknobs across various hospital departments in Japan. The study aimed to evaluate the effectiveness of ATP bioluminescence in detecting contamination on frequently touched surfaces in non-critical areas. Researchers assessed six types of doorknobs—grip, lever, push, insert, and two pull-type variants—located in diverse settings such as staff lavatories, break rooms, linen closets, and dirty utility rooms. Sampling included ATP assays alongside microbial colony detection using the stamp agar method to assess both organic residue and bacterial presence [13].

Results showed significant variation in ATP values depending on the knob design and location. Grip-type and push-type knobs in high-traffic, contamination-prone areas such as utility rooms exhibited significantly higher ATP readings [13]. In contrast, knobs in lower-traffic locations like break rooms showed relatively lower bioburden. The study demonstrated that ATP assays are valuable not only in critical care but also in monitoring hygiene compliance in general hospital environments. It concluded that cleaning protocols should consider usage patterns and environmental exposure to effectively reduce contamination [13].

Additionally, Shimoda et al. [14] explored how the material properties of surfaces influence ATP bioluminescence readings, a critical consideration for the accuracy of ATP-based cleanliness assessments. Conducted in a Japanese university hospital, the study assessed surfaces composed of various materials, including stainless steel, plastic, wood, and fabric, across departments frequently accessed by patients and healthcare workers [14].

The findings revealed statistically significant differences in ATP values between different surface types. Rough and absorbent materials, such as fabric, retained more organic residue and consequently showed higher ATP readings [14]. In contrast, smoother, non-porous materials like stainless steel and plastic yielded lower ATP values. These differences were attributed to the surface’s ability to harbor and retain biological material [14]. The authors emphasized that ATP readings should be interpreted with an understanding of the sampled surface material, recommending the development of material-specific benchmarks to improve the reliability of cleanliness assessments in healthcare environments [14].

In a different context, Phyu et al. [15] evaluated disinfection effectiveness in family medicine offices using ATP measurements taken both after Environmental Services’ morning cleaning and after end-of-day terminal cleaning by medical staff. The results revealed disparities in cleanliness quality depending on the cleaning personnel, underlining the importance of consistent monitoring across shifts [15].

### ICU and terminal room cleaning

The SHINE trial by Ziegler et al. [2] involved 12 ICUs in the United States and evaluated the impact of integrating ATP-based cleaning audits into terminal cleaning protocols. This cluster-randomized study compared standard visual inspections to enhanced ATP-guided feedback mechanisms. Over a 9-month period, ICUs implementing ATP bioluminescence monitoring reported significantly fewer MDRO-positive environmental cultures and a reduction in patient colonization with MRSA and VRE [2]. Notably, surfaces assessed using ATP showed 60% lower bioluminescence values post-cleaning compared to controls, emphasizing its utility in improving cleaning efficacy and promoting staff accountability through direct feedback [2].

Supporting these findings, Chan MC et al. [16] conducted a two-phase prospective intervention study in the cardiology and medical ICUs of a large medical center in Taiwan to assess a new cleaning protocol using ATP assays. In Phase I, only 43.9% of 221 tested surfaces met the cleanliness threshold. Following the implementation of a revised protocol and staff training, Phase II showed a significant improvement, with 76.9% of surfaces passing the ATP-based cleanliness criteria, highlighting enhanced compliance and cleaning effectiveness [16].

Similarly, Hung IC et al. [17] introduced a multimodal approach combining fluorescent markers, ATP bioluminescence assays, and aerobic colony counts (ACC) at an academic medical center. Their prospective study demonstrated that surfaces classified as “totally clean” by the fluorescent marker had significantly lower ATP and ACC values than those deemed partially clean or unclean. This underlined the benefit of real-time, multimodal feedback in reinforcing environmental cleaning practices [17].

Evidence from resource-limited settings further affirms the utility of ATP assays. Dramowski et al. [18] evaluated cleaning efficacy in 25 pediatric ICU isolation rooms at Tygerberg Children’s Hospital in South Africa using ATP bioluminescence, bacterial cultures, and fluorescent markers. They found significant reductions in aerobic colony counts and ATP readings post-cleaning, alongside improved removal of fluorescent markers following staff feedback. This underscores ATP’s role in identifying and addressing cleaning deficiencies even where resources are constrained [18].

Ettenberger et al. [19] extended ATP application to non-traditional ICU equipment, specifically musical instruments used in music therapy services. In their prospective cohort study, both ATP and microbiological assessments before and after cleaning confirmed that the disinfection protocol significantly reduced microbial contamination. These results advocate for the inclusion of ATP monitoring for adjunct therapeutic tools in ICU environments [19].

In a quasi-experimental study, Li et al. [20] assessed the influence of varying chlorine-based disinfectant concentrations on cleanliness outcomes in an ICU setting. Their analysis showed no significant differences in ATP or bacterial counts across concentrations, suggesting that disinfectant strength alone does not guarantee cleaning success. This reinforces the importance of objective surface cleanliness monitoring through ATP testing [20].

Addressing daily, rather than terminal, cleaning, Deshpande et al. [21] employed ATP assays to monitor persistent contamination in ICU environments. Their findings highlighted that areas deemed clean by visual inspection often harbored residual biological material, undetectable without ATP analysis. These results support incorporating ATP into routine daily cleaning audits for comprehensive hygiene management [21].

Finally, Johani et al. [22] provided a microbial ecological assessment of ICU surfaces, revealing that 61% were highly contaminated with biological soil, as indicated by ATP readings. Interestingly, ATP contamination did not always align with bacterial DNA detection via qPCR, illustrating that ATP measures broader organic contamination. The presence of biofilms and resistant organisms further emphasized the need for integrating ATP with microbial surveillance tools for robust environmental monitoring [22].

### Instrument reprocessing and endoscopy units

ATP bioluminescence has emerged as a valuable tool for monitoring the effectiveness of cleaning and reprocessing medical instruments, particularly in high-risk environments such as endoscopy units and surgical departments. A growing body of evidence highlights its role in identifying residual organic material that may escape visual detection, thereby enhancing patient safety and infection prevention.

Chan et al. [7] conducted a comprehensive evaluation across three major endoscopy units in Hong Kong to assess ATP bioluminescence as a verification tool for manual cleaning of flexible endoscopes. Over 600 endoscope lumens were tested immediately after manual cleaning but before automated reprocessing, with 200 RLUs set as the threshold for cleanliness [7]. The results showed that 18% of the samples exceeded this threshold, indicating substantial residual contamination despite visual confirmation of cleaning. These findings demonstrated the limitations of traditional inspection methods and emphasized the need for objective, real-time testing to validate cleaning efficacy [7].

Similarly, Masia et al. [8] investigated the utility of ATP monitoring in a tertiary care hospital in Italy, focusing on 150 reusable surgical instruments post-manual cleaning and pre-sterilization. Their cross-sectional study revealed that ATP values were significantly influenced by the geometric complexity of the instruments. Items such as clamps, scissors, and suction tips, which contain narrow or textured surfaces, retained higher levels of bioburden [8]. The study concluded that ATP testing could serve as a crucial checkpoint in the sterilization workflow, especially for instruments at higher risk of cleaning failure [8].

Expanding upon this concept, Xiong et al. [23] implemented a quality control module integrating ATP bioluminescence in a hospital’s Disinfection Supply Center (DSC). The initiative significantly increased the rate of cleaning qualification for precision surgical instruments, demonstrating the benefit of systematic ATP monitoring in improving overall reprocessing outcomes [23]. In a complementary study, Zhan et al. [23] evaluated ophthalmic phacoemulsification handpieces using both ATP assays and borescope imaging. This dual-modality approach identified internal debris not visible during routine inspection and linked these residues to postoperative inflammatory complications, reinforcing the clinical relevance of comprehensive cleanliness verification [23].

Barakat and colleagues contributed extensively to this field through a series of investigations centered on gastrointestinal endoscopes [24, 25]. In one study, Barakat et al. [24] compared manual and automated drying methods for endoscope channels, using ATP bioluminescence to assess post-drying contamination. Automated drying with the DriScope Aid was found to be significantly more effective, yielding lower ATP values and reducing fluid retention [24]. In a related investigation, Barakat et al. [25] found that the use of simethicone during endoscopic procedures resulted in persistent ATP signals despite full reprocessing, raising concerns about residue retention and necessitating enhanced cleaning protocols.

Further reinforcing the value of ATP testing, Barakat et al. [26] employed a high-resolution inspection endoscope alongside ATP assays to examine the interior surfaces of working channels. Despite minimal visible damage, ATP readings varied across instruments, highlighting residual contamination that visual inspection alone failed to detect [26]. This finding emphasized ATP bioluminescence’s unique role in identifying hidden risks within reprocessed equipment.

Gillespie et al. [27, 28] applied ATP testing to evaluate cleanliness across all steps of duodenoscope reprocessing—precleaning, manual cleaning, and high-level disinfection (HLD). While ATP levels decreased progressively, they often remained above acceptable limits, particularly in elevator-containing duodenoscopes [27, 28]. This raised important concerns about the completeness of reprocessing in complex instruments.

Sethi et al. [29] built upon this by assessing rinsates from suction-biopsy and elevator channels post-HLD. Their study confirmed that ATP values frequently remained elevated even after high-level disinfection, especially in duodenoscopes, contributing to the growing body of evidence on the limitations of current reprocessing methods and the potential infection risks posed by residual contamination [29].

Finally, Obee et al. [30] were among the first to demonstrate the real-time application of ATP bioluminescence in endoscope decontamination management. Their study in two UK hospitals showed that ATP provided rapid, objective assessments of cleanliness and enabled timely corrective interventions when standards were not met [30].

Together, these studies present a compelling case for the integration of ATP bioluminescence into standard reprocessing protocols. Beyond its role in validating cleanliness, ATP monitoring supports quality assurance, enhances staff accountability, and ultimately contributes to safer clinical practices, especially in scenarios where visual inspection or microbial cultures may be insufficient.

### Dental and ophthalmic settings

ATP bioluminescence has emerged as a pivotal tool for evaluating surface cleanliness and verifying infection control protocols in dental and outpatient clinical settings. Offering rapid, objective, and quantitative assessment of organic contamination, ATP assays frequently outperform traditional visual inspections and even culture-based methods.

In pediatric dentistry, Dworkin et al. [31] conducted a randomized controlled trial involving 60 children undergoing restorative procedures to compare the efficacy of Isovac^®^ versus high-volume evacuation (HVE) in reducing aerosol and surface contamination. ATP bioluminescence was used to measure residual contamination on dental chairs and operator personal protective equipment (PPE). The Isovac^®^ group demonstrated a 45% reduction in mean RLU values, confirming its superior ability to limit localized contamination and reinforcing the utility of ATP monitoring in procedural optimization [31].

Expanding on contamination mapping during dental procedures, Watanabe et al. [32] used ATP assays to evaluate the spread of aerosol and splatter across operator and patient surfaces. The study revealed markedly elevated ATP levels post-procedure, emphasizing the limitations of current protective barriers and the critical role of real-time hygiene surveillance [32]. In a complementary study, Watanabe et al. [33] applied ATP bioluminescence to monitor bacterial contamination in dental unit waterlines (DUWLs). They found a strong correlation between ATP readings and culture-based microbial counts, validating ATP as a rapid and reliable method for DUWL monitoring.

In orthodontics, ATP testing has been effectively used to assess biofilm levels and caries risk. Leeper et al. [34] utilized ATP bioluminescence to quantify plaque accumulation in patients prone to white spot lesions. They found higher ATP values in patients with increased sugar consumption and poor oral hygiene, reinforcing the assay’s value in patient-specific risk assessment and biofilm control [34].

Prosthodontic research has also benefitted from ATP applications. Baba et al. [35] compared mechanical versus combination denture-cleaning methods and found significantly lower ATP values and greater patient satisfaction in the combination group, highlighting the assay’s utility in improving hygiene protocols for oral appliances.

In dental microbiology, ATP bioluminescence has supported antimicrobial evaluation. Moon et al. [36] and Choi et al. [37] investigated the effectiveness of N-acetylcysteine in disrupting endodontic biofilms. Both studies reported substantial reductions in ATP levels post-treatment, demonstrating the compound’s efficacy and ATP testing’s usefulness for evaluating anti-biofilm strategies [36, 37].

Beyond dentistry, ATP assays have shown relevance in general outpatient environments. Phyu et al. [15] evaluated the effectiveness of cleaning protocols in family medicine clinics by measuring ATP levels following morning cleaning by Environmental Services and evening terminal cleaning by clinical staff. Their results revealed variability in cleanliness based on personnel, underscoring the need for standardized cleaning protocols supported by objective verification methods like ATP monitoring [15].

In ophthalmology, Zhan et al. [38] conducted an observational study at a Chinese center to assess contamination within phacoemulsification handpieces using ATP bioluminescence and borescope imaging. Despite external cleaning, over 25% of instruments showed elevated internal ATP levels, particularly after prolonged use, demonstrating the necessity for lumen-specific cleaning validation in precision surgical tools [38].

Collectively, these studies underscore the indispensable role of ATP bioluminescence in enhancing infection prevention and quality assurance across dental, outpatient, and ophthalmological care. From assessing surfaces and instruments to monitoring waterlines and complex lumens, ATP assays offer an efficient, standardized, and highly sensitive approach to maintaining hygiene in clinical practice.

### Veterinary applications

ATP bioluminescence has emerged as an essential tool for hygiene assessment in veterinary settings, offering rapid, objective, and quantitative insight into surface contamination—far surpassing the limitations of traditional visual or culture-based inspections. Its applications range from disinfection monitoring in clinical facilities to biosecurity management in agricultural environments, making it a practical method for real-time evaluation of cleaning effectiveness across diverse animal care contexts.

At the University of Minnesota Veterinary Medical Center, Akinyede et al. [9] conducted a six-month cross-sectional hygiene audit using ATP bioluminescence. The study focused on high-risk areas such as surgical suites, diagnostic laboratories, and animal holding rooms [9]. Frequent contamination was identified on anesthesia carts and touchscreen monitors—sites often overlooked during routine cleaning [9]. These findings led to revised cleaning protocols and targeted staff training, demonstrating ATP testing’s role in both identifying gaps and supporting infection prevention education.

In agricultural settings, Johnson et al. [39] evaluated the cleanliness of farrowing rooms in a commercial sow farm after pressure washing. ATP assays provided immediate feedback on hygiene status, confirming whether disinfection was sufficient before reintroducing animals [39]. This rapid verification supports timely biosecurity decisions and limits pathogen transmission.

A similar approach was adopted by Iovane et al. [40] in buffalo milking parlors in Italy. Their study revealed seasonal variations in bacterial contamination through ATP and culture-based analyses, allowing for seasonally adjusted cleaning strategies [40]. Meanwhile, Van Driessche et al. [41] applied ATP luminometry to feeding equipment in dairy farms, correlating ATP values with environmental and calf health parameters. These findings highlighted ATP’s utility not only in surface hygiene monitoring but also in indirectly supporting animal health outcomes.

ATP bioluminescence has also been used to assess trailer sanitation. Letsch et al. [42] demonstrated that visual inspection alone was insufficient for ensuring livestock trailer cleanliness after commercial washing. ATP testing identified residual contamination invisible to the naked eye, reinforcing its importance in controlling disease transmission across transportation systems [42].

Beyond hygiene, ATP has found applications in veterinary parasitology. Nguyen et al. [43] used ATP assays to evaluate the viability of Haemonchus contortus larvae, validating bioluminescence as a proxy for biological activity and expanding its role in veterinary research and antiparasitic testing.

Collectively, these studies affirm that ATP bioluminescence is a highly versatile and effective tool in veterinary practice. It enhances quality assurance in clinical and agricultural settings, supports antimicrobial stewardship through improved disinfection oversight, and opens new avenues for research applications. As veterinary medicine places increasing emphasis on biosecurity, zoonotic disease prevention, and animal welfare, the routine use of ATP-based monitoring is positioned to become a cornerstone of best practices in hygiene management.

### Comparison with visual inspection

ATP bioluminescence has emerged as a critical tool for assessing cleanliness in clinical, veterinary, and food safety settings. In contrast to traditional visual inspection, which is subjective and limited to detecting only gross contamination, ATP assays provide quantitative, objective, and rapid feedback on residual organic matter. A growing body of evidence highlights the limitations of relying solely on visual evaluation.

While visual inspection is easy to perform and cost-effective, it suffers from low sensitivity and often fails to detect invisible residues or microbial contamination. Frota et al. [44, 45] showed that visual inspection frequently overestimated cleanliness when compared to ATP bioluminescence and microbiological testing, particularly on high-touch clinical surfaces.

Chen et al. [3] performed a direct comparison between visual inspection, performance observation, and ATP testing in Taiwanese hospitals. Their evaluation of over 500 hospital surfaces revealed that more than 30% of those rated as “clean” by visual inspection exceeded 250 RLUs, and 15% yielded positive cultures, indicating microbial presence [3]. These findings demonstrate the inadequacy of visual inspection in detecting residual contamination and underscore the objectivity that ATP monitoring offers.

Similarly, Huang et al. [46] used visual inspection, ACC, and ATP assays to evaluate surface cleanliness at a medical center. Their results showed that ATP bioluminescence was significantly more sensitive than visual methods, especially in detecting microbial contamination following terminal cleaning procedures [46].

Luick et al. [47] supported these findings by showing that both ATP bioluminescence and fluorescent markers had greater diagnostic accuracy than visual inspection when benchmarked against aerobic cultures—the microbiological gold standard.

Furthermore, Nante et al. [48] emphasized that ATP assays provide real-time feedback and allow for immediate corrective actions, giving them a distinct advantage over retrospective visual audits.

In a modern example from ophthalmology, Zhan et al. [38] combined ATP testing with borescope imaging to assess contamination in phacoemulsification handpieces. They reported that invisible internal debris, often missed during visual checks, was detected through ATP and endoscopic assessment—highlighting the need for objective tools in complex device reprocessing [38].

Finally, Mildenhall and Rankin [49] emphasized that while visual inspection remains useful for identifying gross debris or spills, it is insufficient as a standalone measure in settings that demand high levels of hygiene assurance.

### Comparison with microbial cultures

ATP bioluminescence and microbial culture methods are widely used techniques for assessing hygiene in clinical and therapeutic environments. While both aim to detect contamination, they differ substantially in their methodology, turnaround time, sensitivity, and practical utility [44, 45].

ATP bioluminescence detects residual organic matter by measuring ATP—a universal marker found in all living cells. Importantly, ATP assays capture both microbial and non-microbial residues such as blood, mucus, and food particles. The primary advantage of ATP testing is its ability to provide rapid, quantitative results, making it a powerful tool for real-time hygiene monitoring in high-risk settings such as ICUs, operating rooms, and even non-traditional environments like music therapy services [44, 45].

Microbial cultures, by contrast, are considered the gold standard for detecting viable pathogens. These methods identify live microorganisms based on their ability to grow on selective media under controlled conditions. However, cultures are time-intensive—requiring 24–72 h—and may underestimate contamination if microorganisms are in a viable but non-culturable state or if sample handling is suboptimal [44, 45].

Numerous studies have directly compared these two modalities. Ettenberger et al. [19] evaluated musical instruments used in ICUs, applying both ATP bioluminescence and microbial cultures before and after disinfection. While microbial cultures often yielded negative results, ATP levels significantly decreased only after thorough cleaning, demonstrating ATP’s superior sensitivity in detecting residual organic material [19].

Similarly, Kim et al. [11] used ATP assays during outbreak investigations in a neonatal intensive care unit (NICU). While ATP testing rapidly identified high-touch surfaces with potential contamination, microbial cultures were required to confirm the presence of specific pathogens such as Enterococcus faecium and Staphylococcus capitis, reinforcing the complementary nature of both approaches [11].

Sethi et al. [29] further illustrated ATP’s utility in duodenoscope reprocessing. Their study revealed elevated ATP values even when microbial cultures were negative, suggesting either residual non-viable biological material or organic debris. This highlighted ATP’s broader detection range, but also a key limitation—its inability to distinguish between viable pathogens and inert organic matter [29].

In orthopedic operating rooms, Richard and Bowen [50] found that ATP assays identified contamination on surfaces that visual inspection and microbial cultures had overlooked, reinforcing the notion that ATP is more sensitive in routine environmental monitoring.

Additionally, Hung et al. [20] adopted a human-factors engineering approach in a Taiwanese hospital to compare ATP monitoring with traditional culture-based evaluations. When ATP feedback was incorporated into environmental services workflows, failed surface rates dropped by 50%, and cleaning compliance significantly improved—especially during staff retraining periods [20]. This study demonstrated how ATP monitoring not only improves surface hygiene detection but also acts as a behavioral reinforcement tool, strengthening institutional infection prevention efforts [20].

In summary, ATP bioluminescence offers unmatched speed and sensitivity for assessing organic contamination, making it ideal for routine surveillance and immediate process feedback. Meanwhile, microbial cultures remain essential for pathogen identification and confirmatory diagnostics. Used together, these complementary methods provide a robust framework for environmental hygiene monitoring, infection control, and patient safety assurance in diverse healthcare settings.

### Comparisons with other rapid tests

Tang et al. [51] conducted a study in food service areas of hospital canteens in Hebei, China. They compared the effectiveness of ATP assays and traditional coliform paper tests on kitchen utensils. ATP testing detected contamination on 32% more samples than coliform methods [51]. Their analysis indicated that ATP was more responsive to organic residues, even when microbial loads were below culture thresholds [51].

Puig-Collderram et al. [52] applied ATP bioluminescence in a novel context: assessing the synergistic activity of antibiotic combinations against XDR Pseudomonas aeruginosa isolates. Their microbiological assay used ATP output as a surrogate marker for bacterial viability [52]. Findings demonstrated that ATP reduction closely mirrored time-kill curve data, supporting ATP as a proxy for pharmacodynamic interactions.

### Role in outbreak investigations

ATP bioluminescence has emerged as a critical tool in outbreak investigations, providing real-time, actionable insights into surface contamination that are not available through traditional microbiological techniques alone. During infectious disease outbreaks, the rapid identification of contamination hotspots is essential for containing transmission. ATP bioluminescence enables immediate evaluation of residual organic material—microbial or otherwise—allowing for prompt intervention and improved infection control.

Kim et al. [53] investigated a Serratia marcescens bloodstream infection outbreak in an obstetric ward for high-risk pregnant women. Despite no patients having central venous catheters, ATP bioluminescence testing revealed elevated contamination levels (> 1000 relative light units) in high-touch areas and medication preparation zones. These findings pinpointed hygiene failures invisible to visual inspection, prompting targeted disinfection that curtailed potential transmission sources [53].

Similarly, Kim et al. [11] conducted a detailed environmental surveillance study during two consecutive NICU outbreaks caused by Enterococcus faecium and Staphylococcus capitis. ATP testing of over 300 surfaces and devices revealed high bioluminescence signals clustered around neonatal incubators and infusion pumps. Immediate interventions, including equipment replacement and enhanced cleaning protocols, led to a 68% reduction in ATP levels and cessation of new outbreak cases over a six-month follow-up period [11].

Hardy et al. [54] extended the utility of ATP bioluminescence in the context of increased Clostridium difficile infections. Their investigation showed that surfaces deemed clean by visual inspection often had elevated ATP values, revealing hidden contamination. Incorporating ATP monitoring led to stricter hygiene protocols and improved surface cleanliness during the outbreak period [54].

Johani et al. [22] further contributed to this evidence base by mapping ATP levels on ICU surfaces and correlating them with biofilm presence. Their work emphasized the assay’s potential in identifying persistent microbial reservoirs that require intensified decontamination strategies [22].

Additionally, Watanabe et al. [55] demonstrated ATP bioluminescence as a valuable visualization tool in long-term care facilities prone to outbreaks. By highlighting contamination trends in shared environments, ATP feedback helped tailor cleaning practices based on contamination risk [55].

Collectively, these studies illustrate the indispensable role of ATP bioluminescence in modern outbreak response. While microbial cultures remain essential for identifying specific pathogens, ATP testing excels at rapid risk identification, real-time process evaluation, and guiding immediate environmental hygiene interventions. Their combined application provides a comprehensive and dynamic framework for outbreak prevention and control.

### Advances in ATP-based technology

ATP bioluminescence technology has undergone significant advances in recent years, broadening its application across clinical, environmental, and food safety domains. Traditionally used for rapid surface hygiene assessments, new developments have pushed the boundaries of ATP-based monitoring into more specialized and dynamic areas such as airborne pathogen detection, live bacterial identification, and antimicrobial susceptibility testing.

Airborne bioaerosol surveillance has benefited notably from ATP-based methods. Liu et al. [56] reviewed recent innovations in combining air sampling with ATP bioluminescence for the quantitative detection of airborne microbes. These technologies allow for real-time assessment of bioaerosol burden in healthcare and public settings, providing a rapid alternative to slow-growing culture methods [56]. Complementary reviews by Zhang et al. [57] and Sajjad et al. [58] further supported these advances, highlighting how methodological improvements, including sample preconcentration and ATP signal amplification, have enhanced sensitivity and reliability in detecting microbial biomass in ambient air.

Beyond air monitoring, ATP biosensing has evolved into a sophisticated analytical platform for microbial diagnostics. Cao et al. [5] developed a portable ATP biosensor capable of detecting live Escherichia coli O157:H7 in food samples. This system utilized bacteriophage-modified magnetic stir bars and a bio-proliferation amplification step to enrich ATP from viable pathogens, achieving detection limits below 10 CFU/mL [5]. Although originally designed for food safety applications, the sensor’s design holds translational potential for clinical pathogen surveillance, especially in resource-limited environments where culture-based diagnostics may not be feasible [5].

In another significant leap, Sever et al. [10] introduced an ATP-consumption-based platform for rapid antimicrobial susceptibility testing. Their system measured the reduction in bioluminescent signal as bacteria responded to antibiotic exposure. Remarkably, this method provided accurate susceptibility results within two hours and demonstrated 95% concordance with conventional minimum inhibitory concentration (MIC) testing [10]. This innovation underscores ATP’s growing role in rapid diagnostics and precision antimicrobial stewardship, offering an efficient means to guide early, targeted therapy during infection management.

Collectively, these studies underscore a transformation in ATP-based bioluminescence from a hygiene tool into a versatile diagnostic and surveillance technology.

### Limitations and variability in readings

ATP bioluminescence is widely used for rapid surface hygiene assessment across clinical, food safety, and environmental settings due to its speed and real-time feedback. However, its utility as a standalone tool is limited by several important factors.

A major limitation is its lack of specificity—ATP assays detect both microbial and non-microbial residues, such as food particles, mucous, and blood. This can lead to false positives. Mildenhall and Rankin [49] emphasized that ATP does not differentiate viable pathogens from inert organic matter, making it an indirect proxy for cleanliness. Moreover, ATP testing is destructive, preventing longitudinal viability monitoring, as highlighted by Bian et al. (2025) in organoid research [59].

Environmental and technical variability also limit reliability. Bakke [60] and Sanna et al. [61] observed that results can be influenced by surface type, humidity, disinfectant residues, and operator technique, sometimes overestimating cleanliness compared to microbial culture. Tršan et al. [6] reported significant fluctuations in ATP readings over time in a hospital cleanroom despite uniform cleaning, pointing to external factors like humidity and surface texture as confounders.

Device-dependent discrepancies are another concern. Xu et al. [62] demonstrated inconsistent ATP values across detectors under the same conditions. Liao et al. [63] further noted that bioaerosol ATP readings vary with particle size and sampler efficiency, complicating interpretation. Sajjad et al. [58] and Nante et al. [48] called for standardized protocols and defined thresholds to enable valid cross-setting comparisons.

While ATP is a valuable process control tool, particularly for identifying gross contamination or guiding immediate action, its limitations—non-specificity, environmental sensitivity, lack of standardization, and cost—require it to be used as part of a broader, multimodal hygiene monitoring strategy incorporating microbial cultures and visual or fluorescent assessments.

## Discussion

The findings of this comprehensive review underscore the widespread utility of ATP bioluminescence in monitoring surface hygiene across a range of healthcare and related environments. ATP assays offer a rapid, objective, and practical method for evaluating cleanliness, particularly on high-touch surfaces where traditional visual inspection often fails. In general hospital settings, ATP monitoring consistently revealed residual contamination even after routine cleaning, highlighting the inadequacy of visual assessment alone. Its integration into daily workflows allowed for real-time feedback, which in turn enhanced staff awareness and compliance with hygiene protocols.

In ICUs and high-risk environments, ATP testing emerged as a powerful adjunct to terminal cleaning processes. Studies demonstrated that ATP monitoring significantly reduced microbial loads on surfaces and was linked to lower rates of colonization with multidrug-resistant organisms. Its effectiveness was amplified when combined with staff education, fluorescent markers, or microbial cultures, promoting a culture of accountability and reinforcing proper disinfection practices. This evidence positions ATP as both a process verification tool and a behavioral reinforcement mechanism within infection prevention strategies.

Beyond traditional clinical spaces, ATP assays proved essential in monitoring the cleaning efficacy of complex reusable medical instruments such as endoscopes, duodenoscopes, and phacoemulsification handpieces. These tools are particularly vulnerable to incomplete decontamination due to intricate designs. ATP bioluminescence allowed for early identification of residual organic material, enabling timely intervention prior to sterilization. In some cases, ATP testing revealed contamination invisible to both visual inspection and standard microbial cultures, confirming its role in safeguarding patient safety through enhanced quality assurance in instrument reprocessing.

In dental, ophthalmic, and outpatient settings, ATP assays facilitated contamination mapping during and after procedures. These environments often lack standardized surface auditing protocols, and ATP provided a consistent, quantitative benchmark for assessing cleanliness. Moreover, its application extended to dental unit waterlines, orthodontic appliances, and prosthodontic devices, where real-time feedback helped refine cleaning protocols and reduce biofilm burden. Importantly, ATP also identified inconsistencies between different cleaning personnel, pointing to the need for standardization across shifts and care providers.

Veterinary and agricultural applications further showcased the assay’s versatility. From surgical suites to livestock transport trailers, ATP testing identified contamination hotspots and supported biosecurity decision-making in animal care. The assay’s rapid turnaround enabled timely corrective action, and its correlation with animal health indicators highlighted its indirect value in preventing zoonotic transmission. These findings expand the relevance of ATP bioluminescence beyond human medicine and into One Health frameworks focused on intersectoral hygiene management.

Despite its benefits, ATP bioluminescence is not without limitations. Its inability to distinguish between viable pathogens and inert organic matter, susceptibility to environmental and material variability, and lack of global standardization necessitate cautious interpretation. Device-dependent variability and the influence of disinfectant residues or surface texture can affect reliability. Thus, ATP should not be used in isolation but rather integrated into a multimodal hygiene monitoring strategy that includes microbial cultures, fluorescent markers, and visual inspection. By leveraging the strengths of each method, healthcare systems can achieve more comprehensive and effective infection control.

## Conclusion

ATP bioluminescence has emerged as a rapid, practical, and increasingly validated method for assessing surface cleanliness in diverse healthcare environments. As demonstrated across a wide range of studies, including large trials and cross-sectional evaluations in ICUs, operating rooms, dental clinics, and veterinary centers, ATP testing consistently outperforms visual inspection and frequently detects organic residues even after routine cleaning. Its immediate feedback facilitates real-time interventions, supports staff training, and contributes to lowering microbial contamination rates—especially for high-touch and high-risk surfaces.

Despite its advantages, ATP bioluminescence is not without limitations. Variability between luminometers, differences in surface materials, and the absence of standardized RLU thresholds challenge its reproducibility across institutions. Furthermore, ATP readings detect organic matter but not specific pathogens and thus should complement rather than replace microbial cultures in certain contexts, such as outbreak investigations or reprocessing validation. Emerging technologies, such as ATP-based biosensors and antimicrobial susceptibility platforms, indicate promising expansions of its utility beyond surface hygiene monitoring. Moving forward, standardized protocols, detector calibration, and integration with other surveillance methods are essential for fully leveraging the diagnostic and preventive potential of ATP bioluminescence in healthcare infection control.

## Data Availability

The data supporting this review are available within the article.
